# Quality evaluation of dried tomato fruit and optimization of drying conditions using a modified solar dryer integrated with an automatic solar collector tracker

**DOI:** 10.1038/s41598-025-89248-x

**Published:** 2025-03-05

**Authors:** Abdallah Elshawadfy Elwakeel, Awad Ali Tayoush Oraiath, Mohsen A. Gameh, Ahmed S. Eissa, Samy F. Mahmoud, Mohamed Hamdy Eid, Atef Moussa, Mostafa B. Mostafa, Mohamed Farag Taha, Samah A. T. Abulmeaty, Aml Abubakr Tantawy

**Affiliations:** 1 Agricultural Engineering Department, Faculty of Agriculture and Natural Resources, Aswan University, Aswan, 81528 Egypt; 2 Department of Agricultural Engineering, Faculty of Agriculture, Omar Al Mukhtar University, P.O. Box 991, Al Bayda, Libya; 3 Soils and Water Department, Faculty of Agriculture, Assiut University, Assiut, 71526 Egypt; 4 Agricultural Products Process Engineering Department, Faculty of Agricultural Engineering, Al-Azhar University, Cairo, 11751 Egypt; 5 Department of Biotechnology, College of Science, Taif University, Taif City, Saudi Arabia; 6 Institute of Environmental Management, Faculty of Earth Science, University of Miskolc, 3515 Miskolc-Egyetemváros, Hungary; 7 Geology Department, Faculty of Science, Beni-Suef University, Beni-Suef, 65211 Egypt; 8 Water and Farm Irrigation Systems Engineering Department, Faculty of Agricultural Engineering, Al-Azhar University, Cairo, 11751 Egypt; 9 School of Agricultural Engineering, Jiangsu University, Zhenjiang, 212013 China; 10 Department of Soil and Water Sciences, Faculty of Environmental Agricultural Sciences, Arish University, Arish, 45516 North Sinai Egypt; 11 Food Science and Technology Department, Faculty of Agricuture, Al-Azhar University, Cairo, Egypt; 12 Food Science and Technology Department, Faculty of Agriculture and Natural Resources, Aswan University, Aswan, 81528 Egypt

**Keywords:** PV system, Solar dryer, IoT, Automated system, Tomato fruit, Dehydration, Color quantification, Plant sciences, Plant development

## Abstract

In the current study, a modified solar dryer (SD) integrated automatic solar collector tracker (ASCT) was used for drying tomato fruit (TF) at three slice thicknesses of 4, 6, and 8 mm on both drying systems at three air speeds of 1, 1.5, and 2 m/s until reaching the equilibrium moisture content. Where the comparison study was conducted between the ASCT, and another SD integrated with a fixed solar collector (FSC). The obtained results of the current study showed that the maximum solar intensity and ambient air temperature during the test period were 900 W/m^2^, and 43.6 °C, respectively. As well as the highest efficiency of the PV system was 16.69% at the same time. On the other hand, the height, greatest diameter and smallest diameter of the TF used in the current study ranged between (4.6 and 5.2 cm), (3.5 and 4.2 cm), and (3.4 and 4.1 cm), respectively. As well as both the athematic and geometric diameters ranging between 3.87 and 4.47 cm and 2.26 and 2.38 cm, the sphericity values of tomatoes tend to have a round shape. In addition, the obtained results that there was no significant effect of the hot air velocities on the drying time, but the final moisture content (MC) decreased with increasing the hot air velocities. The lowest final MC was 6%, and it was recorded with a slice thickness of 4.0 mm that dried on SD integrated with ASCT. Additionally, color analysis showed that, the darkest tomato slices were dried on the SC integrated with FSC at a hot air velocity of 1.0 m/s. Meanwhile, the intensity of red and yellow colors significantly increased after drying with the SD integrated with ASDT. Furthermore, the chemical analysis of the dried tomato slices showed that the highest rehydration ratio of 4.43 kg water/kg dry matter was obtained for the dried tomato slices dried on SD integrated with ASCT at a hot air velocity of 1.5 m/s and a slice thickness of 8.0 mm. As well as the highest values of pH were monitored on the dried tomato slices on ASCT in comparison to the other tomato slices dried on FSC. Also, the highest ascorbic acid content recorded was 141 mg/100 g (d.b.) in tomato slices with an 8.0 mm thickness, dried using SD combined with FSC at an air velocity of 2 m/s. After drying, the total phenols content increased in all dried tomato samples but decreased with lower hot air velocities.

## Introduction

Tomatoes *(Lycopersicon esculentum*) are among the most widely cultivated and consumed crops globally, with an annual production of 186.11 million tonnes ^1–3^. Egypt is the fifth-largest producer, contributing 6.25 million tonnes each year. Tomatoes can be eaten fresh or processed into various products, such as juice or canned goods, and are rich in essential nutrients, including vitamins B6, C, and A ^1,3–10^. However, they have a high-water content, making up about 96% of their total weight ^11,12^. The demand for processed tomato products has grown substantially, especially in the retail and food ingredient sectors ^13^. Drying is a common method used to preserve tomatoes in agricultural and industrial settings ^14,15^. It is considered one of the earliest forms of food preservation, even predating cooking. Drying involves removing moisture from food to produce a product that can be stored safely for long periods ^16–19^. This process also decreases the product’s weight and volume, resulting in cost savings for storage and transportation ^20^.

Thermal processing alters the tomato’s color, sensory, nutritional, and functional properties ^21^. Researchers used many drying techniques for drying agricultural products including industrial dryers operated with fossil fuels, microwave drying, infrared drying, heat pump drying, solar-geothermal drying, heated greenhouses, and freeze drying are utilized to reduce these changes during drying ^6,22^. Solar dryers (SD) offer a viable alternative to the reliance on fossil fuels by utilizing solar energy for the drying process. These dryers are an effective technology for preserving food and promoting environmental sustainability ^14,15^. The SD consumes less energy and incurs lower operational expenses than alternative drying technologies. Solar energy is widely available in numerous locations and represents a sustainable and environmentally friendly power source. Industrial dryers have gained popularity in recent years because of the limitations of conventional sunlight dryers in drying agricultural products. The utilization of industrial dryers frequently results in diminished product quality, shortened shelf life, and optimized health issues ^6,23,24^. Industrial dryers, primarily utilized in industrialized nations, are often not feasible for small-scale operations in developing regions due to their high capital and operational costs ^25^.

Additionally, many researchers have used SDs to prompt the quality of dried tomatoes, such as, Hossain et al. ^25^ developed a hybrid SD integrated with a flat-plate concentrating collector for drying tomatoes. A comparison was made between the performance of the hybrid SD and an open sun-drying system. The drying effectiveness was assessed based on the drying rate, lycopene content, ascorbic acid content, color, and total flavonoid content. The characteristics of solar-dried tomatoes surpassed those of open sun-dried tomatoes. Nabnean et al. ^26^ evaluated the performance of a newly designed SD for drying cherry tomatoes. The dried tomatoes were fully protected from rain and insects, producing high-quality dried products. Al Maiman et al. ^27^ investigated the effects of solar drying on the storability, physicochemical properties, and antioxidant potential of dried tomatoes. This method enhanced the storage potential and physicochemical attributes of tomato slices, extending their shelf life by up to six months. Owureku-asare et al. ^28^ used a custom passive mixed-mode SD to study dried tomatoes’ dehydration and microbiological quality. The results showed a reduction in aerobic mesophile counts in solar-dried tomatoes compared to those dried in the open sun, making solar-dried tomato powder safer for consumption. Eze and Ojike ^29^ compared the open sun drying method with various types of SDs, revealing that SDs were more effective at preserving vitamins A and E. Sharma et al. ^30^ found that the rehydration ratio, shrinkage, taste, and hardness tests demonstrated that the indirect spray drying method produced higher quality dried tomato flakes than the open sun drying technique.

In previous literature, various authors proposed different SDs with different SCs, such as greenhouse system ^31^, solar concentrators ^32^, evacuated-tube air collector ^33^, flat plate solar collectors (FPSCs) ^34–39^. FPSCs are commonly used in solar drying systems ^40^. The advantages of this particular collector encompass its affordability, lack of requirement for solar tracking systems, simple construction, and little maintenance expenses ^41^. FPSCs have several limitations, primarily because their performance is dependent on the quantity of solar radiation they receive. The efficiency of these systems can be significantly impacted if they are not optimally positioned to capture sunlight. To address this, a mechanical tracking system that follows the movement of the sun and adjusts the solar panels to maintain a perpendicular angle to the sun’s rays would greatly enhance the quantity of radiation captured by the solar collectors (SC) ^42^. Additionally, various efforts have been made to enhance the thermal efficiency (TE) of SC. ElGamal et al. ^43^ developed a SD combined automatic solar tracking FPSC for drying apple fruit where thermal efficiency increased by 45% compared to fixed FPSC. Bhowmik and Amin ^44^ used a solar reflector to increase the solar collecting system’s thermal efficiency by about 10%. Zheng et al. ^45^ increased the thermal efficiency of the SC up to 60.5% by using a compound parabolic concentrator SC. Zou et al. ^46^ used a small parabolic SC improved its TE to 67%. Chamsa-ard et al. ^47^ reported a TE of 78% by employing a SC coupled with a heat pipe and evacuated tube. Similarly, Rittidech et al. ^48^ achieved a 76% TE utilizing a circular glass tube design. Wei et al. ^49^ assessed FPSC to increase the TE to 66%. Verma et al. ^50^ demonstrated a 21.94% increase in TE under forced convection mode compared to a traditional SC. Ramachandran et al. ^51^ used a chaffier concentrator to enhance the TE by 6%, while Ismail et al. ^52^ showed a 15% improvement using a biaxial solar tracker with mirrored reflection. Ndukwu et al. ^19^ developed a natural-convective solar dryer (NCSDR) integrated with sodium sulfate decahydrate (Na_2_SO_4_·10H_2_O) and sodium chloride (NaCl) as thermal storage medium are presented. Ndukwu et al. ^53^ presented a new hybrid solar-biomass dryer and carried out thermal analysis based on energy and exergo-sustainability analysis. Additionally, Ndukwu et al. ^54^ developed a low-cost wind-powered active solar dryer integrated with glycerol as thermal storage.

These studies collectively highlight various approaches to boosting the thermal efficiency of different types of SC. Few studies have focused on improving the TE of FPSCs. A considerable deficiency in existing research pertains to the enhancement and assessment of the operational efficacy of sun-tracking flat solar collectors. Although considerable efforts have been made worldwide to enhance the thermal effectiveness of SC, experimental research in this specific area is still limited. Additionally, there is a notable lack of studies investigating the impact of these SC on the quality of tomato fruits.

The current study aimed to improve the drying efficiency of tomato slices by integrating a SD with an IoT-controlled solar collector tracker and optimizing drying conditions. A key feature of this dryer is its advanced control system, which utilizes IoT technology to track the sun, addressing the limitations of fixed flat plate solar collectors. This innovation resulted in higher and more stable air temperatures inside the drying chamber throughout the day, reduced drying times, and enhanced the quality of the dried tomatoes. The SD was tested at different air velocities of 1.0–2.0 m/s, as well as with tomato slice thicknesses of 4.0–8.0 mm. The quality of the dried tomatoes was assessed based on final moisture content (MC), total phenols (TPs), ascorbic acid (AA), pH, color, acidity, and rehydration ratio (RR).

## Material and methods

### Overview of the drying system

**Fig. 1:**
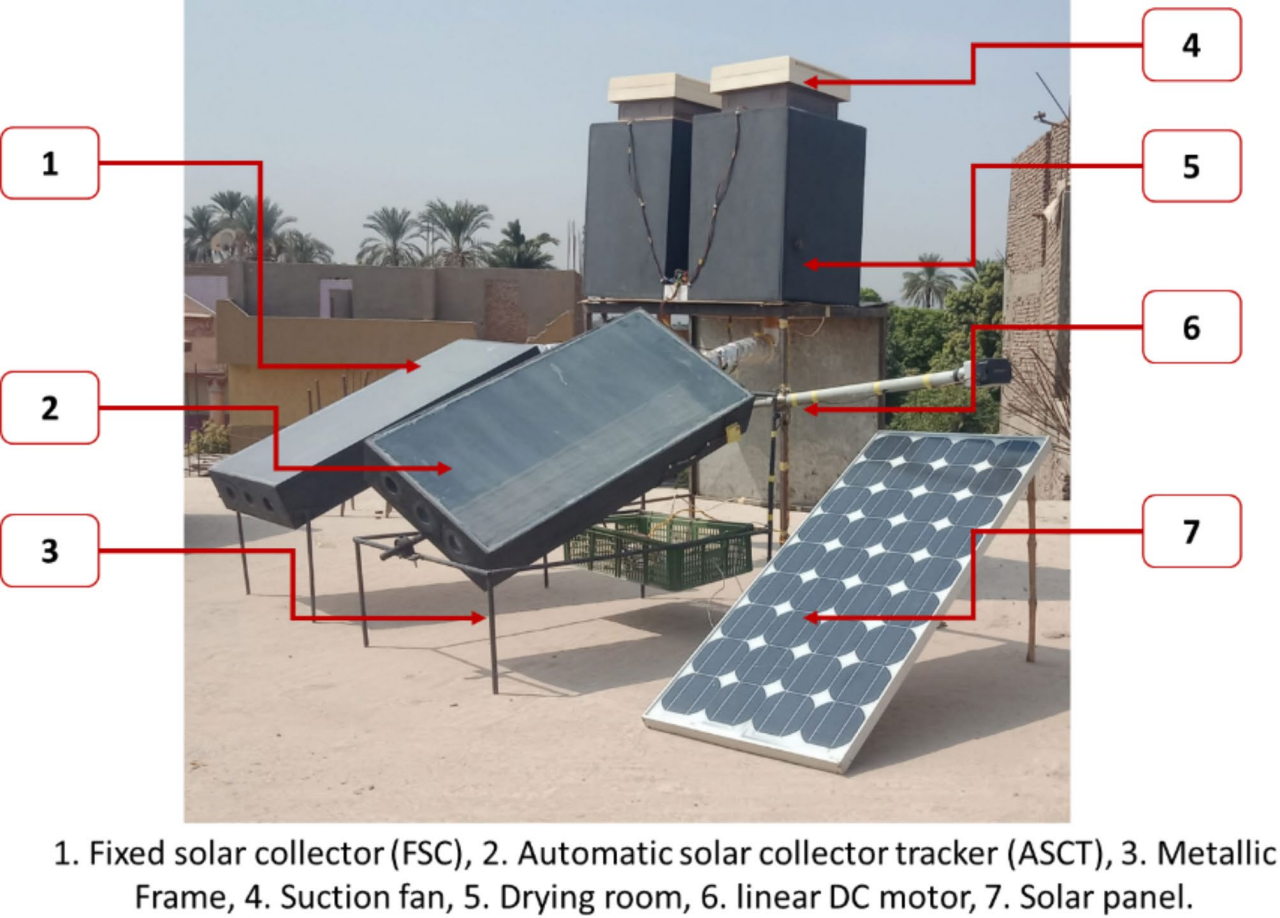
Main components of the drying system ^24,37,38^.

To evaluate the impact of using the modified SD integrated ASCT on dried tomato slices quality, two symmetric SDs were designed in Luxor City, Egypt. One of them is a traditional SD integrated with a fixed solar collector (FSC), where the FPSC was ordinated to the south with a title angle of 28°. The other SD was coupled with an ASCT, where the ASCT was programmed to track the sum movement from east to west (single axle rotation). Both SDs were designed with the same dimensions and manufactured from the same materials. The drying system comprises many components including a SC, drying room, tracking system, suction fan, PV system, and air temperature and relative humidity measuring unit. Figure 1 depicts the key components of the drying system. All design equations and operating systems used for the SD integrated ASCT are comprehensively detailed in Elwakeel et al. ^38^.

#### Solar collector

**Fig. 2:**
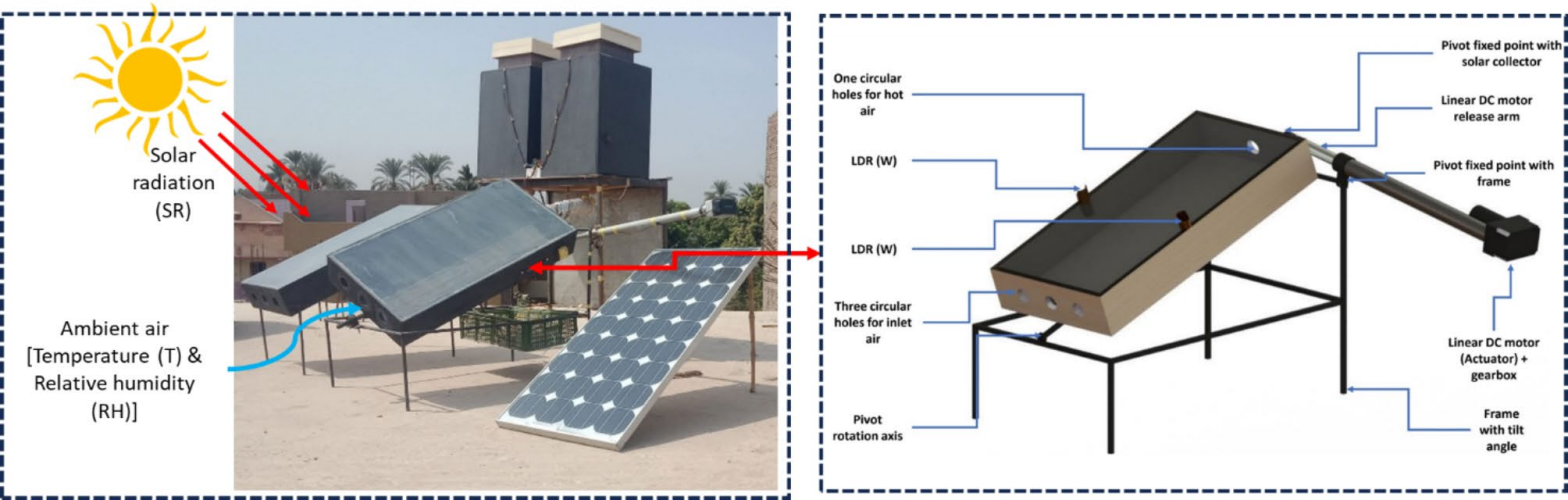
Main components and position of the solar collector ^38^.

Both SCs had dimensions of 100, 50, and 15 cm in length, width, and depth. The absorber plate was manufactured from galvanized metal sheet with 3 mm in thickness. The absorber plate was insulated with wood dust 3 cm in thickness. Both SCs were covered with 3 mm glass cover. The ASCT contains a single-axle solar tracking process. Figure 2 presents the key components and position of the SC.

#### Drying room

The drying room, measuring 0.44 m × 0.63 m, is constructed of wood and features a single drying tray covered with plastic mesh. The tray is equipped with an electronic balance to periodically measure the weight of the dried tomato slices. Additionally, a suction fan is installed at the top of the drying room.

#### Tracking system

The tracking system was designed based on the IoT concept and it combined many electronic parts, as follows; 1. An Arduino board (model: Arduino Uno) was used as a CPU, 2. A two LDR sensors, 3. A four-channel relay kit, 4. A linear actuator (model No: HARL-3624 +) with a maximum operating power of 10 W, operating current ranged between 0.4 – 0.8 A, operating voltage of 36 V, and maximum operating length of the linear motor of 24-inch, 5. A converter (input: 110– 220 V AC; output: 36 V DC 10 A) was used to supply the solar tracking system with the required power. Both LDR sensors were positioned on the upper side of the SC (One LDR sensor on the eastern side (LDR_1_) and the other LDR sensor on the western side (LDR_2_)). The Arduino board received signals from both LDR sensors, if the obtained reading from the LDR_2_ sensor was higher than the obtained reading from the LDR_E_ sensor (LDR_2_ ≥ (LDR_1_ ± 5 Lux)), the linear actuator will operate and rotate the SC from east to west (clockwise), if else, the linear DC motor (anticlockwise) to rotate the SC from west to east (in the morning).

An electrical circuit specifically designed for this investigation was utilized to assess the temperature and relative humidity of the surrounding air during the field experiments. The electronic circuits consist of many electronic parts, such as an Arduino board (model: Mega 2560), and a dry humidity and temperature sensor (model: DHT-22). The solar radiation data were obtained from the weather station in Luxor, Egypt.

Prior to usage, the light intensity sensors (LDR) were validated by calibrating them with standard devices. This involved comparing the readings of the LDR sensors with those acquired from a digital light meter (LUX meter) of the UNI-T UT383 model from China. The calibration process involved using an LED light source that emitted varying levels of illumination, ranging from 0 to 1000 lx. Additionally, a digital temperature and humidity meter (model: UT333s), was used to calibrate the temperature and humidity sensor (model: DHT-22). As shown in Table 1 the total uncertainties in the sensors’ reading errors and measurement devices were computed, and the result was ± 1.61%. This value is quite small when compared to the acceptable range of ± 10% as established by Rulazi et al. ^55^.

**Table 1:** The accuracy of the different instruments and sensors was used in the current investigation ^38^.

Parameters	Unit	Instrument	Range	Sensitivity	Resolution	Error, %
Air temperature	°C	DHT-22 sensor	-10 – 80 °C	± 1 °C	0.1 °C	0.1414
Relative humidity	%	DHT-22 sensor	0 – 100%	± 2%	0.1%	0.1414
Solar radiation	W/m^2^	Spectral pyranometers	0–2000 W/m^2^	± 10 W/m^2^	0.1 W/m^2^	0.1414
Weight of dried TF	kg	Electronic digital balance	0.0–50 kg	± 0.020	5 g	0.707
Airspeed	m/s	A digital anemometer	0.0–30 m/s	± 0.1 m/s	0.1 m/s	0.1414
Voltage and current (PV system)	V, A	Digital Multimeter	0.2–1000 V 20 µA-20 A	–	0.01 V 0.01 A	0.01414 0.01414
Light intensity	Lux	LDR sensor	0.0–1000 Lux	± 1 Lux	0.1 Lux	0.1414
Weight of fresh TF	kg	Electronic digital balance	0.0–10 kg	± 10 g	10 g	1.414
Uncertainty, %					± 1.61%	

#### PV system and suction fan

An axial flow suction fan, rated at 40 W and operating at 220 V, was utilized to draw ambient air through the SC and into the drying chamber. This fan was adjustable, allowing for air velocities of 1.0, 1.5, and 2.0 m/s. The fan and other electronic components were powered by a 75-W PV system.

### Experimental procedure

**Fig. 3:**
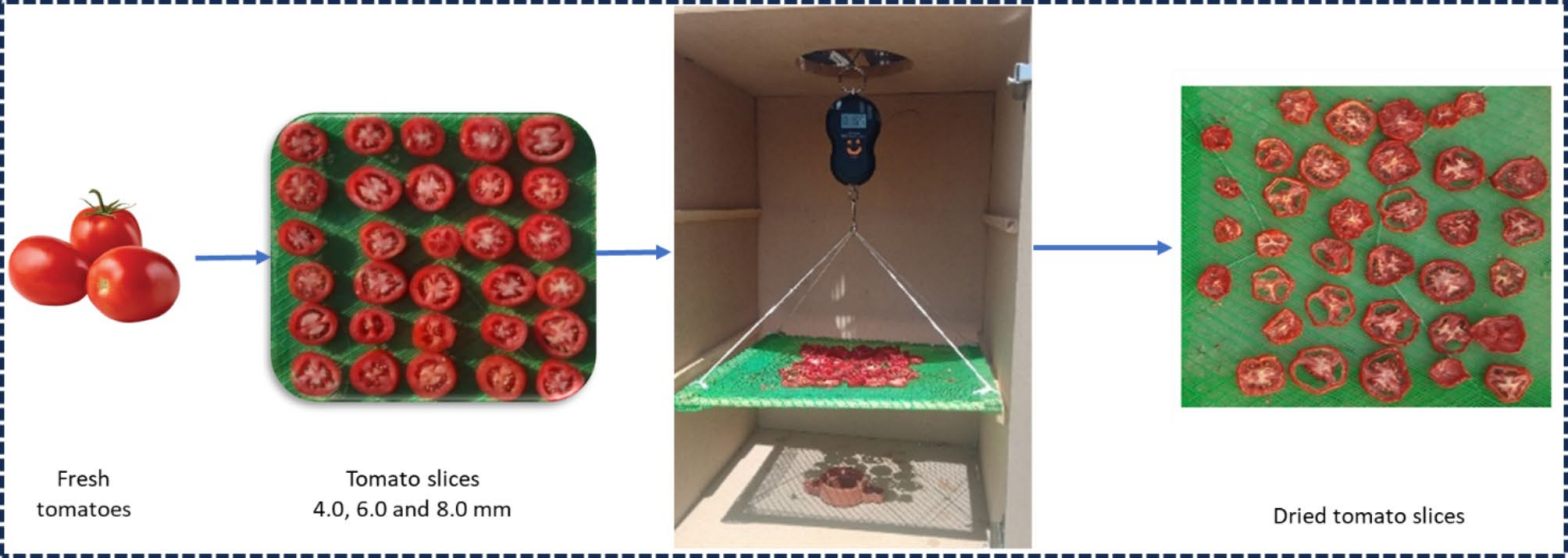
Drying steps of tomato slices ^38,56^.

Tomatoes (*Lycopersicon esculentum*) were sourced from a local market in Luxor City, Egypt. The tomatoes were selected for their uniform color and size, with individual fruits weighing between 80 and 100 g. They were first washed to remove any dust and dirt, then sliced into three thicknesses of 4.0, 6.0, and 8.0 mm utilizing a laser-sharpened kitchen knife. The sliced tomatoes were then arranged on the drying tray. The initial moisture content of fresh tomato slices was 92% ± 2 (w.b.). The drying process continued until the equilibrium moisture content and weight were reached using three air velocities of 1.0, 1.5, and 2.0 m/s. Figure 3 illustrates the steps involved in drying the tomato slices.

### Evaluation of the PV system

The energy input to the PV system (P_in_) was calculated according to Eq. 1. And the energy consumed by the AC suction fan is used as an output source of energy in the calculation of the efficiency of a PV system, according to ^48^, as shown in Eqs. 2 and 3.

1 $${P}_{in}= {A}_{coll}{\int }_{0}^{t}{Ins}_{coll}\left(t\right)dt$$

2 $${P}_{out}= {V}_{oc}\times {I}_{sc}$$

3 $$Efficiency= \frac{{P}_{out}}{{P}_{in}\times FF}$$

where ${V}_{oc}$ is the open-circuit voltage, ${I}_{sc}$ is the short-circuit current, and *FF* is the fill factor.

### Determination of chemical analysis of fresh and dried tomato samples

The chemical analysis of fresh and dried tomato samples was measured in the Laboratory of Food Science and Technology, at Aswan University. Measurements were carried out immediately after purchasing fresh tomatoes, where measurements were carried out under laboratory conditions and at an average air temperature of 30 °C.

#### Assessment of initial moisture content (MC)

The MC on a wet base was estimated using an electric oven at 105 °C for 3 h, using fresh tomato slices with an average weight of 4.0 g, using Eq. 4, as stated by Eke ^57^.

4 $$MC=\left[\frac{{W}_{w}- {W}_{d}}{{W}_{w}}\right]\times 100$$

where ${W}_{w}$ denotes the tomato samples’ wet weight, in grams, and ${W}_{d}$ refers to the of the tomato specimens’ dried weight, also in grams.

#### Ascorbic acid assay

The ascorbic acid (AA) content was assessed following the procedure described by Ranganna ^58^. A 1.0 g sample was extracted with 2% metaphosphoric acid. From this extract, 1.0 mL was mixed with 9 mL of a dye solution containing 2,6-dichlorophenol dye and sodium bicarbonate. The mixture was incubated in the dark for 10 min, after which the absorbance was measured at 515 nm. The AA concentration was estimated using a calibration curve for L-AA.

#### Total phenols assay

Total phenols (TPs) content was estimated using the procedure outlined by Flores et al. ^59^. In this procedure, 0.5 ml of methanolic extract was combined with 5 ml of a 10% (v/v) Folin-Ciocalteu reagent in a test tube. After allowing the mixture to react for 5 min, 4 ml of a saturated sodium carbonate solution was added. The solution was then mixed thoroughly and left in the dark for 15 min. The absorbance was recorded at 765 nm using a UV–visible spectrophotometer, and the results were reported as milligrams of gallic acid equivalent to per 100 g of dry matter.

#### Color quantification

Color quantification of both dried and fresh tomato slices was carried out using a colorimeter (*model: CR-410, Konica Minolta Sensing Americas, Inc., USA*), as stated by ^23,60–63^, and the International Commission on Illumination (ICI) color coordinates L*, a*, and b*.

#### Rehydration ratio (RR)

The RR was estimated using Eq. 5, as described by ^14,15,64,65^. Where the dried tomato slices were weighed using an electronic balance and then soaked for 50 min in distilled water at room temperature (30 °C). After soaking, the excess water was drained for 2 min, and the tomato slices were weighed once more.

5 $$RR=\frac{{W}_{f}-{W}_{i}}{{W}_{i}}$$

where ${W}_{f}$ denotes the tomato slices’ final weight after draining, and ${W}_{i}$ represents denotes the tomato slices’ initial weight before soaking.

#### Acidity and pH

Acidity and pH were estimated on dried and fresh tomato slices, as stated by AOAC ^66^.

### Statistical analysis

Statistical analysis included the coefficient of determination (R^2^), with R^2^ values and significance levels determined at p ≤ 0.001. The means of each parameter were compared between the two types of drying systems across different tomato thicknesses and air velocities using Duncan’s multiple range test at a 5% significance level (SPSS 22, SPSS Inc., Chicago, IL, USA). Mean values with the same letter were considered statistically similar at p ≤ 0.05.

## Results and discussions

### Calibration of measuring sensors

#### Calibration of light intensity sensors (LDR)

**Fig. 4:**
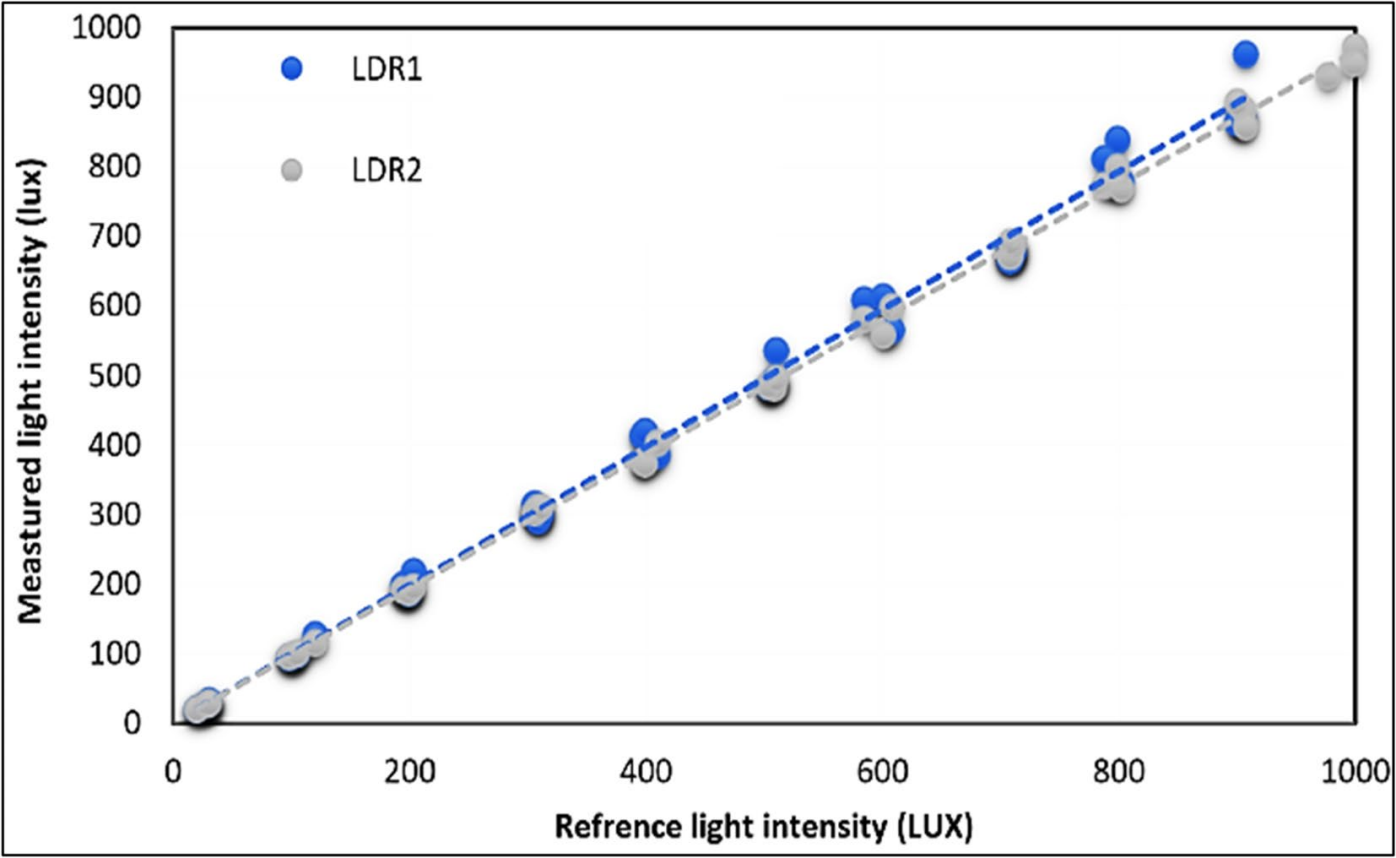
Calibration results for LDR sensors.

Figure 4 shows both measured and reference values for LDR sensors. The calculated R^2^ values were 0.9907 and 0.9653 for both LDR_1_ and LDR_2_, respectively, demonstrating a perfect correlation between the estimated and observed values at p ≤ 0.001. The data acquired from LDR sensors demonstrated excellent performance across numerous light intensities and exhibited strong linear regressions. Specifically, LDR_1_ had a regression equation of y = 0.9907x + 0.7316, while LDR_2_ had a regression equation of y = 0.9653x + 1.1027. Both regressions are closely aligned with the 1:1 line.

#### Calibration of dry humidity and temperature sensors (DHT-22)

**Fig. 5:**
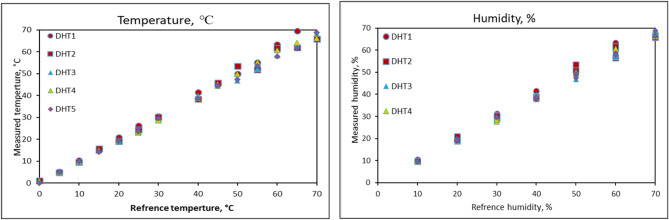
Calibration results of dry humidity and temperature sensors (DHT-22).

The calibration results for the DHT-22 sensor are displayed in Fig. 5. The x-axis was used to plot the reference values of relative humidity and temperature of hot air, while the y-axis was used to plot the measured data. The data obtained demonstrated a robust correlation between the measured and reference values, with a maximum R^2^ of 0.9992 at a significance level (p) of less than or equal to 0.001. The DHT-22 sensor performed exceptionally well when tested at different air temperature values, showing a strong linear regression that closely matched the 1:1 line (y = 0.9661x + 0.0499). Similarly, the sensor exhibited excellent performance when tested at various relative humidity values, with a linear regression that closely aligned with the 1:1 line (y = 0.9643x + 0.1143) at the same point. Table 2 shows the fitting equations and correlation coefficient (R^2^) for different DHT sensors. Where the variable (x) signifies any point along the x-axis. To ascertain the values of both (x) and (y), one must first select a point on the x-axis, subsequently substituting this value into the relevant equation to determine the corresponding (y) value.

**Table 2:** Fitting equations and correlation coefficient (R^2^) for different DHT sensors.

No.	Temperature		Humidity	
	Eq.	R^2^	Eq.	R^2^
DHT 1	y = 1.0272x + 0.1861	0.9977	y = 1.0342x − 3.1230	0.7899
DHT 2	y = 0.9688x + 0.6308	0.9926	y = 0.9667x + 0.7183	0.9902
DHT 3	y = 0.9656x + 0.0684	0.9986	y = 0.9691x − 0.1529	0.9980
DHT 4	y = 0.9801x − 0.0015	0.9975	y = 0.9869x − 3.0957	0.7909
DHT 5	y = 0.9661x + 0.0499	0.9992	y = 0.9643x + 0.1143	0.9988

### Weather conditions during field tests

**Fig. 6:**
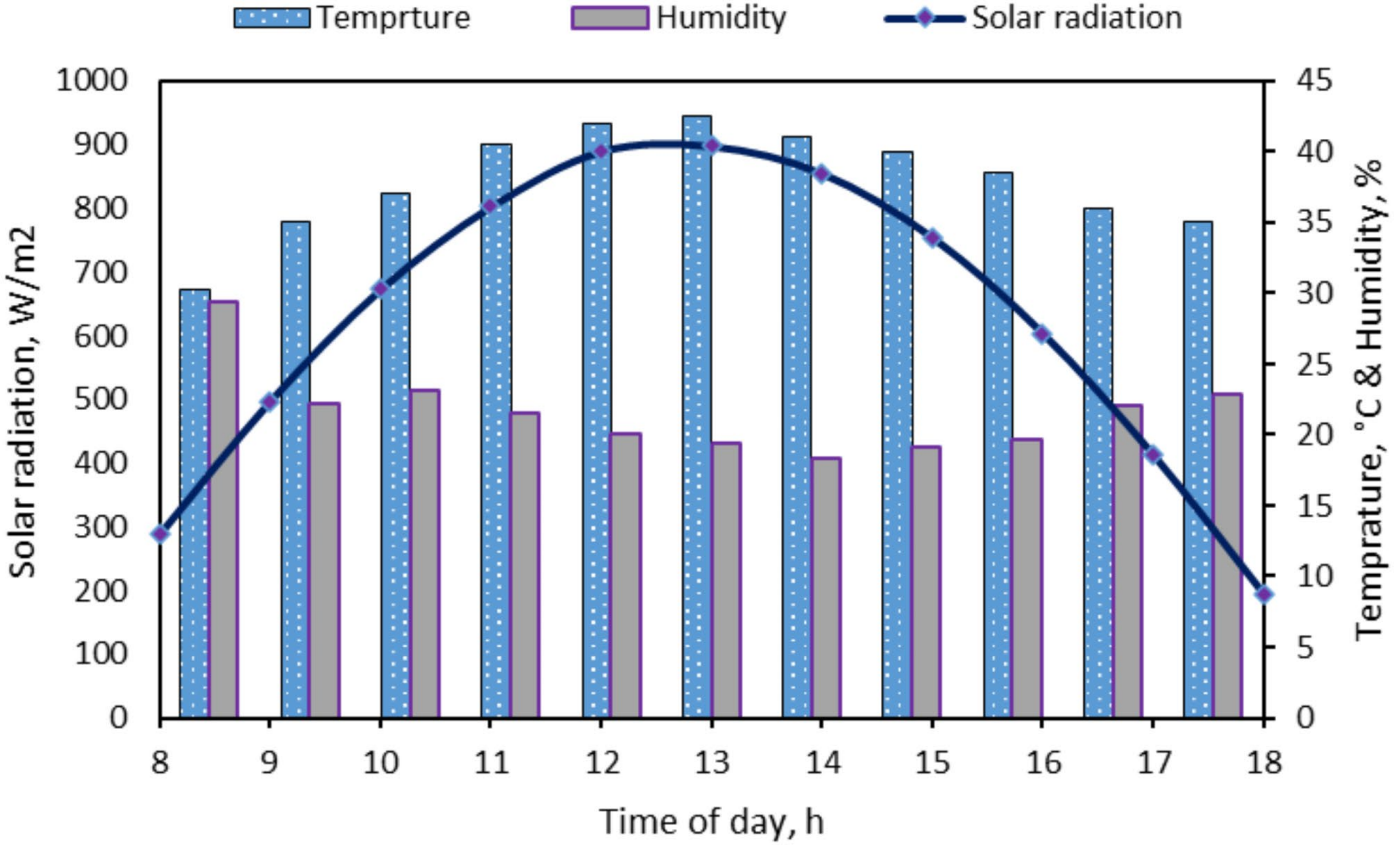
Weather conditions during field tests during the first day of the field tests from 8.00 to 18.00.

Figure 6 illustrates the relative humidity, ambient temperature, and solar radiation throughout the first day of field tests from 8:00 a.m. to 6:00 p.m. The data show that solar radiation gradually increased, peaking at approximately 900 W/m^2^ at 1.0 a.m. The ambient temperature followed a similar trend, reaching a maximum of 43.6 °C at 1.0 p.m. In contrast, the relative humidity exhibited an inverse trend, decreasing as the ambient temperature rose, and reached a minimum of 18.3%.

### Field evaluation of the photovoltaic panel

**Fig. 7:**
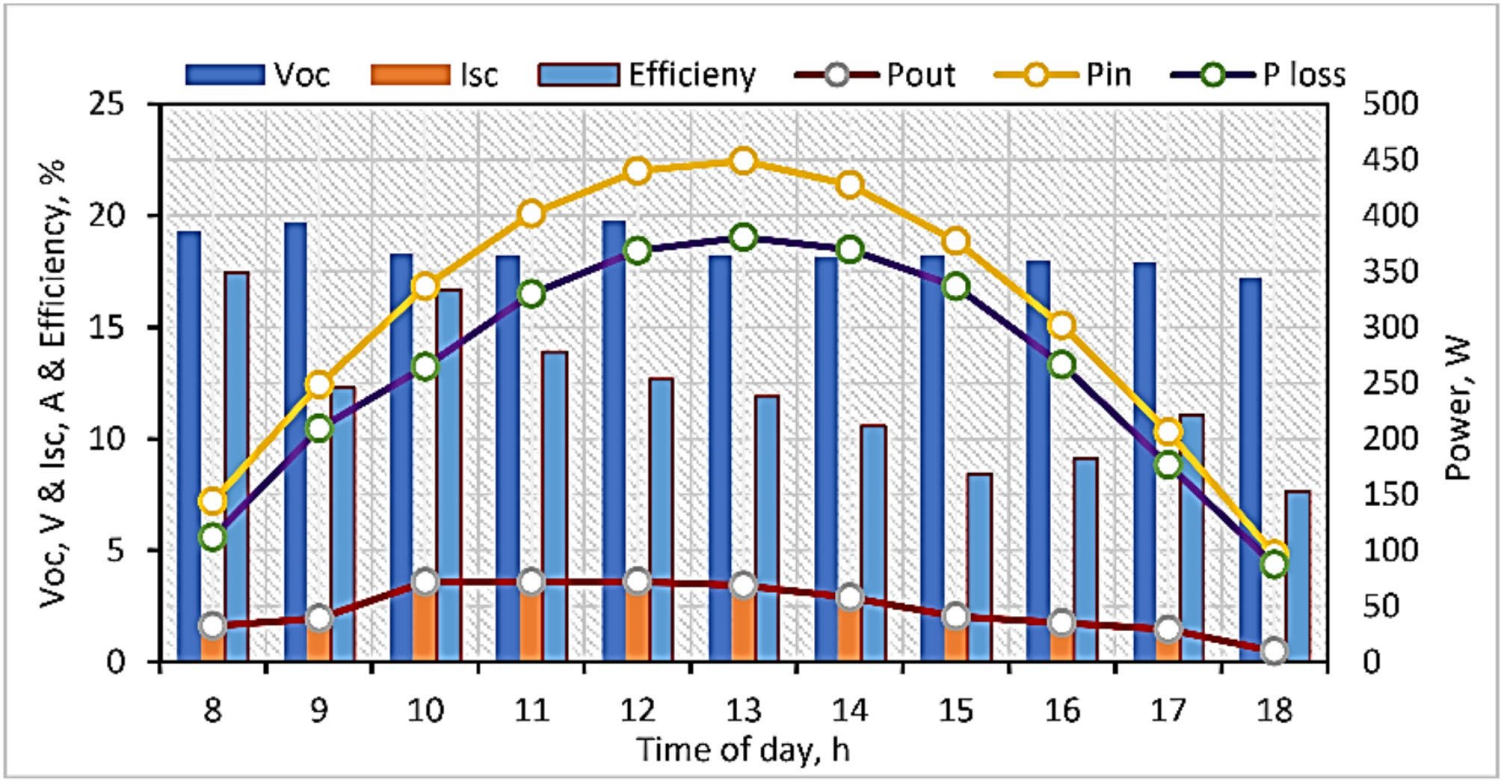
The thermal analysis of the PV system.

As shown in Fig. 7, all electrical parts of the developed SD, such as air circulation fans and the controlling electronic circuit, were operated with a PV system. The PV system consists of a 75-W PV panel, battery charger, battery, and voltage converter. The PV system was oriented southwards in directions 25.6890°N and 32.6975°E. Both open circuit voltage (V_oc_) and shortcut current (I_sc_) were measured on the first day of the field tests by using a digital multimeter, while input power (P_in_), output power (P_out_), power losses (P_loss_), and efficiency of the solar panel were calculated based on the obtained data. The measured data showed that the minimum and maximum V_oc_ and I_sc_ were (17.22 and 19.67 V) and (0.55 and 3.91 A), respectively, and the highest efficiency of the PV system was 16.69%.

### Influence of drying parameters on relative humidity and air temperature inside drying room

**Fig. 8:**
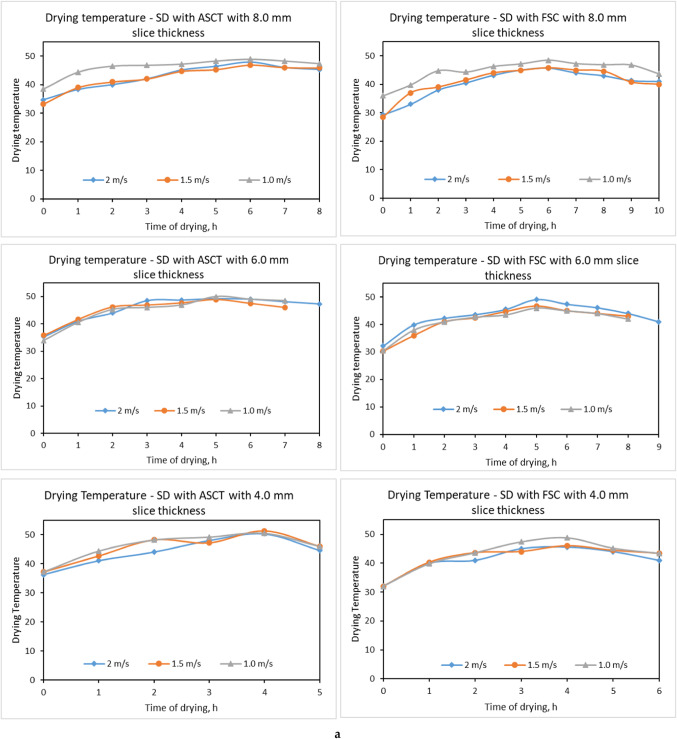

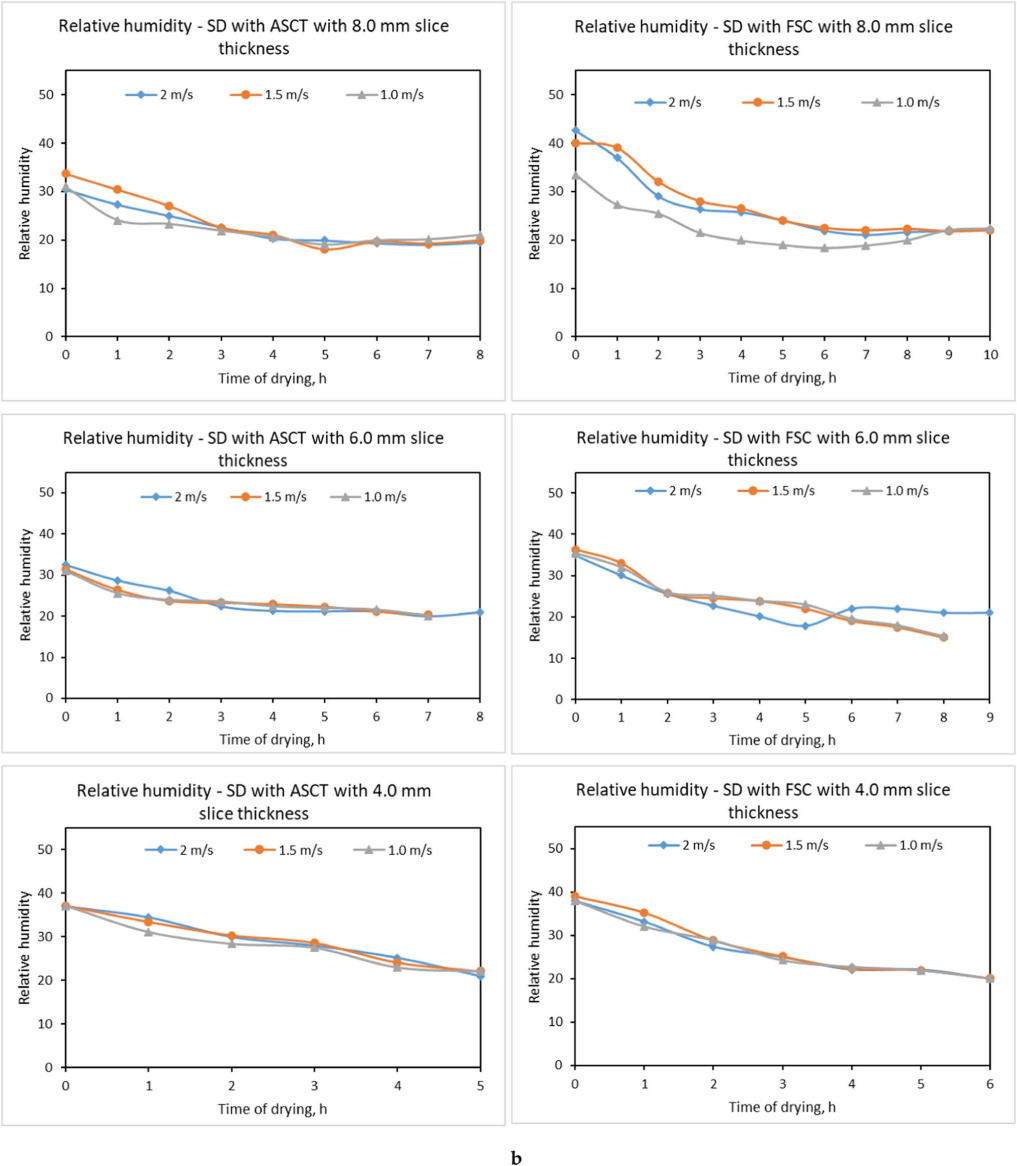
(**a**) Influence of draying parameters on air temperature (°C) and relative humidity inside drying room. (**b**) Impact of draying parameters on air temperature and relative humidity (%) in drying room.

The relative humidity and temperature of the room’s air are very important factors affecting the final MC and the time consumed to achieve the equilibrium MC ^41^. To explore the influence of SC type and hot air velocities on air temperature and relative humidity inside the drying room, three thicknesses of tomato slice were used (4.0 mm, 6.0 mm, and 8.0 mm), in addition to three air velocities (1.0 m/s, 1.5 m/s, and 2.0 m/s) for both SDs integrated with FSC and ASCT. Experimental results indicated that the thickness of tomato slices does not significantly influence the air temperatures within the drying room; however, an increase in slice thickness resulted in a marginal rise in the relative humidity of the air due to the greater volume of water evaporated from the thicker slices, as illustrated in Fig. 8a and b. These results agree with the findings of Metwally et al. ^22,23^ and Elwakeel et al. ^22,23^.

The results presented in Fig. 8b indicate a significant improvement in air temperatures and a reduction in relative humidity inside the drying room of the SD connected to the ASCT, compared to the SD connected to the FSC at various air velocities. This improvement is attributed to the ASCT’s ability to track the sun’s movement throughout the day, allowing it to collect maximum sunlight on the absorber plate. The indoor air temperature in the drying room with the ASCT was approximately 18.43% higher than that in the drying room with the FSC, increasing by about 8.24 °C to reach temperatures between 23.42 °C and the ambient air temperature. The SC achieved a maximum air temperature of 64.2 °C, while the drying room recorded a maximum temperature of 48.9 °C. A SD with an integrated heat energy storage system reached a maximum air temperature of 62.4 °C within the SC ^67^. Nimrotham et al. ^68^ reported that a greenhouse SD attained an average air-drying temperature of 50 °C with a relative humidity of 36%. Koyuncu et al.^69^ noted that greenhouse dryers increased the surrounding air temperature by 5–9 °C. Mutasher ^70^ investigated the effect of a sun-tracking system on the thermal efficiency of SDs, finding a 14% increase in the temperature within the SC compared to stationary systems.

Additionally, it was observed that higher air velocities within the drying room were associated with lower air temperatures. This finding aligns with Abdellatif et al. ^71^, which showed that three identical Quonset greenhouse SDs tested at varying mass flow rates (0.122 kg/s, 0.183 kg/s, and 0.259 kg/s) exhibited increased interior air temperatures as the flow rate decreased. Specifically, the indoor air temperatures rose by 21.9, 12.5, and 9.4 °C, respectively, compared to the outside temperature of 31.6 °C. Furthermore, the relative humidity of the air is inversely correlated with air temperatures, with increased air velocity leading to a slight rise in relative humidity in both types of SDs. These observations are supported by the findings by Yang et al. ^72,73^ and Wangda and Ohsawa ^72,73^.

### Influence of drying parameters on final MC and drying time

**Fig. 9:**
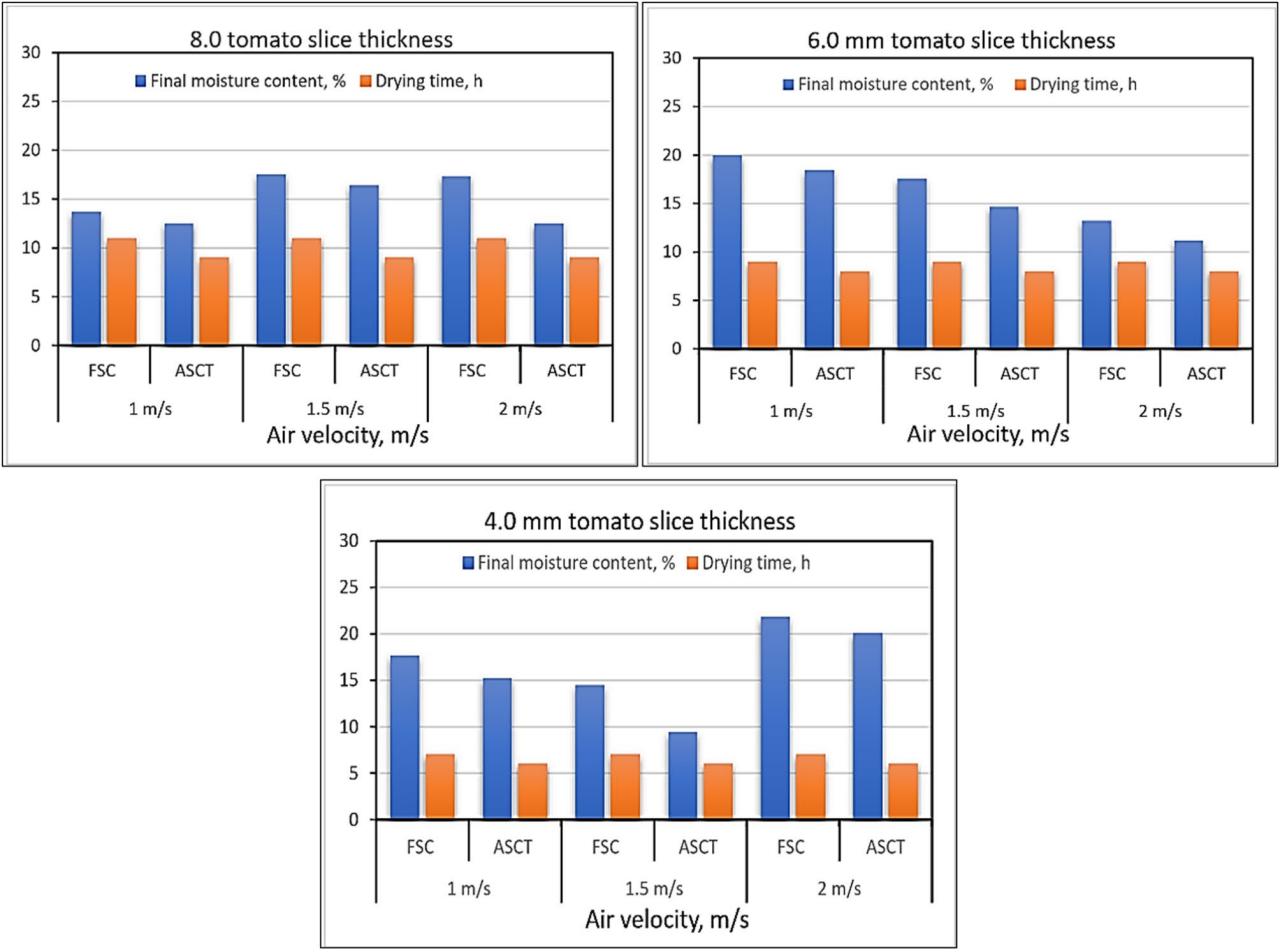
Influence of draying parameters on final MC and drying time of dried tomatoes.

To assess the effects of slice thickness, hot air velocities, and the type of SC on the final MC and drying time of dried tomatoes, two different types of SDs were utilized in this study. Both configurations shared the same overall structure and were made from identical raw materials, but each was equipped with a different drying system. One dryer used a FSC, while the other utilized an ASCT. Experiments were conducted using three slice thicknesses (4.0 mm, 6.0 mm, and 8.0 mm) and three different air velocities (1.0 m/s, 1.5 m/s, and 2.0 m/s). As shown in Fig. 9, there was no significant impact of hot air velocities on drying time based on the SC type. Moisture content was monitored daily at 12 p.m. Although there may be minor variations, they were not substantial enough to be observed clearly. Additionally, since the air velocities used in this study were closely aligned, alternative velocities could be explored in future research. The data presented in Fig. 9 indicates that the final moisture content decreased with increasing hot air velocity. The lowest moisture content for slice thicknesses of 6 mm and 8 mm was recorded at an air velocity of 2 m/s, whereas the lowest moisture content for a slice thickness of 4 mm occurred at an air velocity of 1.5 m/s. Furthermore, drying time was shorter for all slice thicknesses in the SD equipped with ASCT compared to the SD with FSC. Notably, the indoor air temperature in the drying room utilizing ASCT was approximately 18.43% higher than that in the room using FSC.

These results align with the data presented by Putra et al. ^74^, where the investigation was carried out to explore the impact of air temperature and velocity on the rate of evaporation. The findings indicated that elevated air temperature and increased air velocity directly correlate with a higher rate of evaporation. Also, Dorneles et al. ^75^ stated that, the rise in both air temperature and velocity enhances the acceleration of moisture decrease during the operation. Furthermore, Chandramohan ^76^ reported that, the drying rate increases proportionally with higher air temperatures and increased airflow velocities.

In addition, the slice thickness is crucial in decreasing the drying time compared with the different SDs and hot air velocities. As illustrated in Fig. 9, thinner slices resulted in shorter drying times. Specifically, drying times for 4 mm and 6 mm thick slices ranged from 6–7 h and 8–9 h, respectively. Notably, it took 6 h for the moisture content of 4 mm thick tomato slices to drop from 92.5% to 9.41% at an air velocity of 1.5 m/s using the ASCT. In contrast, 8 mm thick slices required 8 h under the same conditions. Therefore, slice thickness significantly impacts drying time (p < 0.05), as indicated in Table 3.

**Table 3:** Analysis of variance (ANOVA) for chemical properties of dried tomato slices (at p ≥ 0.05).

S.v.	df	Sum of squares						
		Rehydration ratio	Ph	Acidity	ascorbic acid	Total phenol	Final MC	Drying time
Air velocity (AV)	2	**0.599**	**0.219**	0.009	**712.333**	154.434	**16.047**	< 0.0001
Slice thickness (ST)	2	**3.172**	0.009	0.011	**340.333**	552.700	**18.657**	**111.000**
Drying system (DS)	1	**3.350**	0.010	0.008	**486.000**	253.067	**87.554**	**24.000**
AV * ST	4	**2.475**	0.118	0.012	4.667	1502.512	**418.834**	< 0.0001
ST * SD	2	0.186	0.067	0.006	**133.000**	931.167	**2.042**	**3.000**
AS * SD	2	0.016	**0.135**	0.007	37.000	310.767	**4.532**	< 0.0001
AV * ST * SD	4	**2.971**	0.099	0.010	16.000	490.601	**20.099**	< 0.0001
Error	36	1.468	0.534	0.152	570.000	8580.140	5.151	1.639

*The significant factors are highlighted in bold.

### Impact of drying parameters on color analysis

**Fig. 10:**
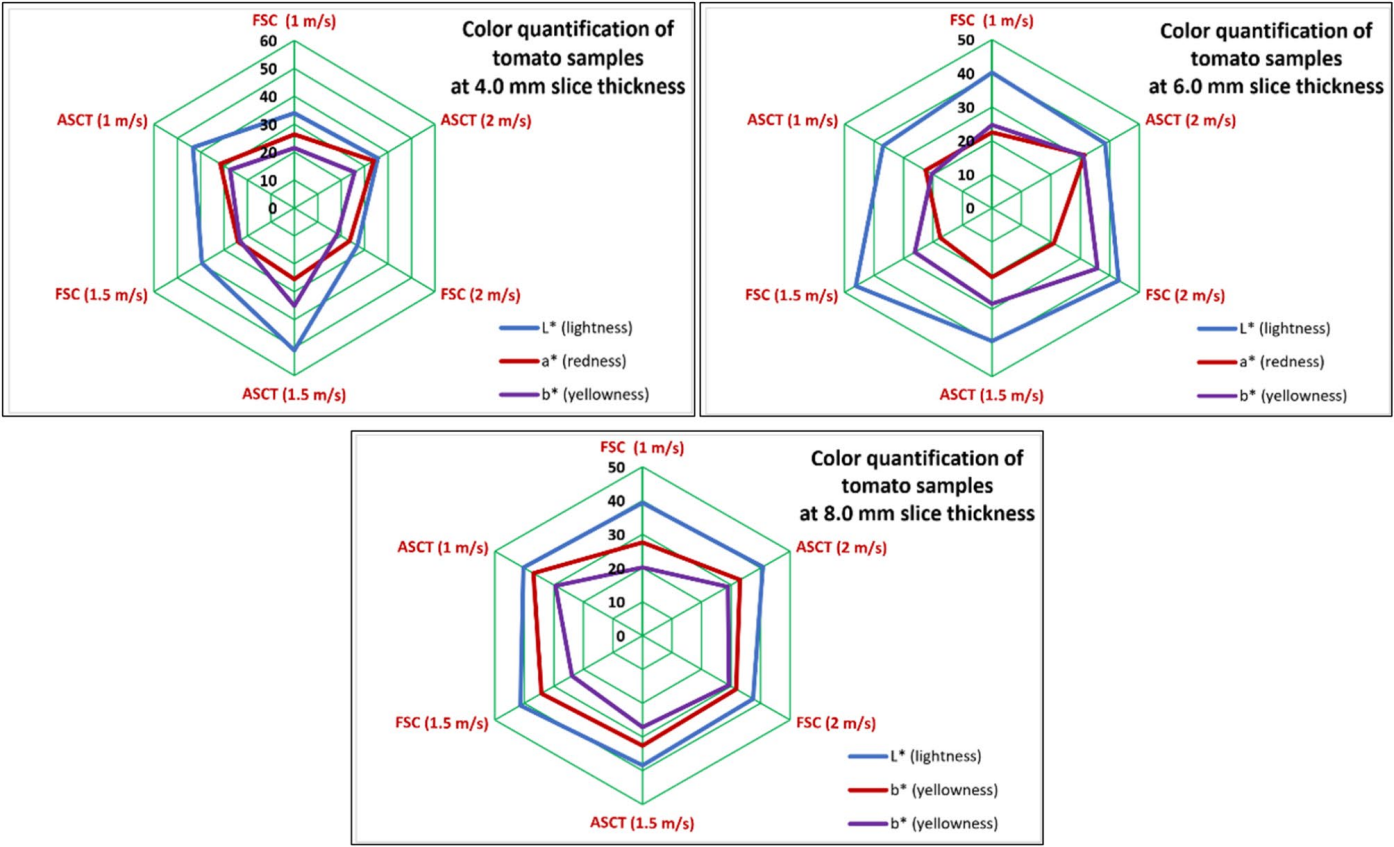
Influence of draying parameters on color qualifications of dried tomatoes.

Color is a critical quality indicator for dried tomatoes, as it significantly influences consumer acceptance of dried foods. Changes in color during the drying process occur due to various chemical reactions. Figure 10 illustrates the L*, a*, and b* values for both fresh and dried tomatoes. Initially, fresh tomatoes had color parameters of L* = 34.32, a* = 30.52, and b* = 23.11. As depicted in Fig. 10, the L*, a*, and b* values changed significantly after drying in both systems. The results indicate that the drying systems, slice thickness, and air velocities all had a notable impact on the final color of the dried tomatoes. Specifically, lightness (represented by L*, a*, and b*) increased with higher air velocities, with the ASCT system producing greater values than the FSC system. This can be attributed to the longer drying times, which resulted in more pronounced changes in the L* value.

These results align with Ashebir et al.^77^, which noted a reduction in color intensity in tomato slices dried at various thicknesses and air velocities. Additionally, it was noticed that darkening intensified as drying air temperature increased, and moisture content remained high. Also, Das Purkayastha ^78^ reported a decline in the L-value of dried tomato slices with rising drying temperatures.

Additionally, the darkest tomato slices were dried in the SC integrated with FSC at a hot air velocity of 1.0 m/s. Meanwhile, the intensity of red and yellow colors significantly increased after drying with the SD integrated with ASDT. These findings come in agreement with the presented data by Arslan and Ӧzcan ^79^ and Izli and Isik ^80^. Furthermore, it is important to note that the findings by Das Purkayastha et al. ^78^ support the current data, as the authors also reported a decline in the 'a*' and 'b*' parameter of tomato slices during drying. This reduction was particularly pronounced in slices dried near the middle and at the dryer outlet, likely due to higher temperatures in these areas.

### Influence of drying parameters on rehydration ratio (RR)

**Fig. 11:**
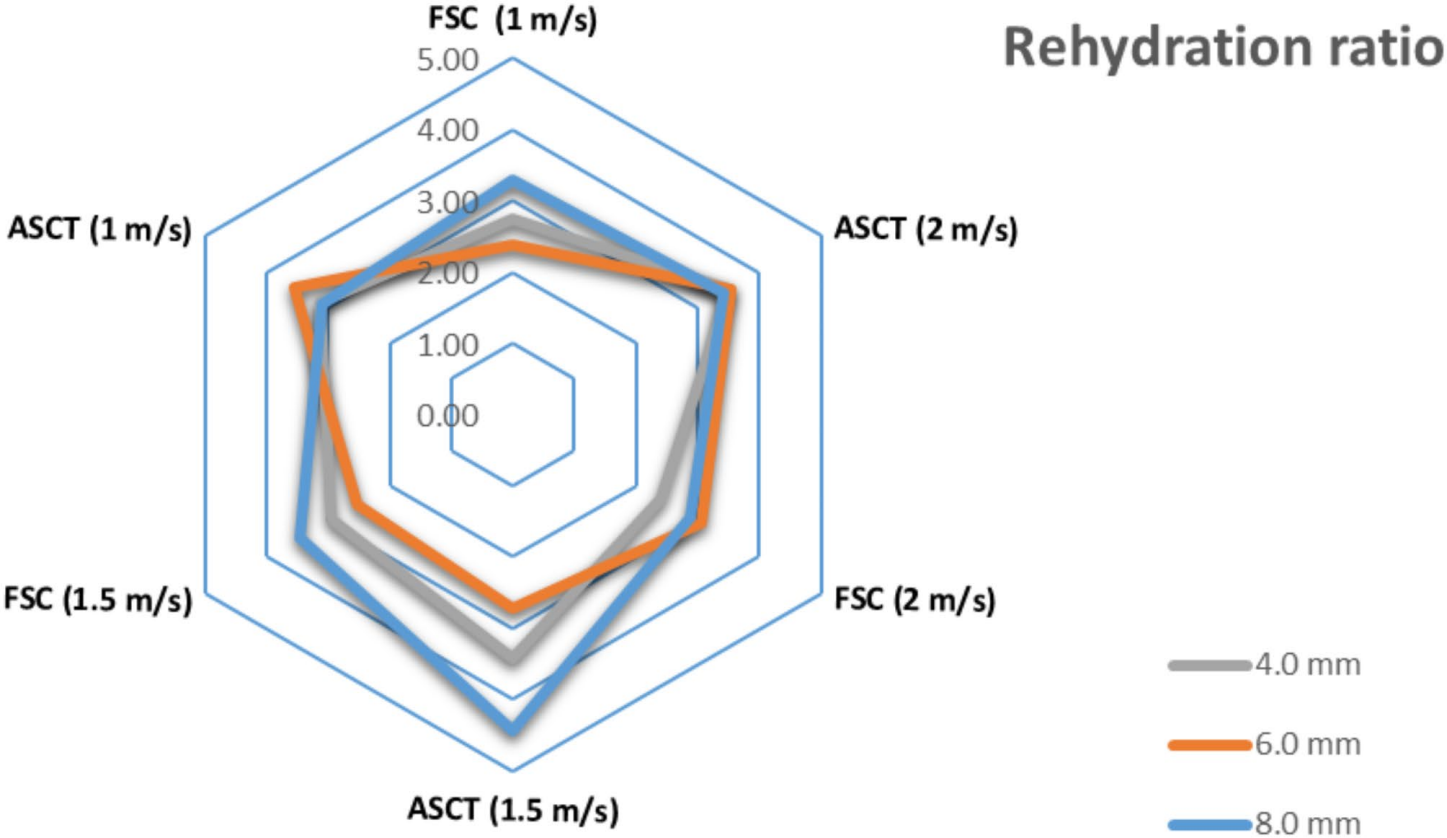
Influence of draying parameters on RR of dried tomatoes.

The RR of water from dried tomato slices was determined at air velocities ranging from 1.0 to 2.0 m/s. The highest RR of 4.43 kg of water per kg of dry matter was achieved with dried tomato slices processed using a SD integrated with an ASCT at a hot air velocity of 1.5 m/s, with the slices being 8.0 mm thick. In this study, the RR values varied from 2.39 to 4.43, as illustrated in Fig. 11, which agrees with Doymaz ^20^ who stated that the RR values of dried tomatoes were lower than 4.5. Alternatively, Rajkumar et al. ^81^ reported that RR values ranged from 2.9 to 3.2 for sun-dried tomato slices. Dufera et al. ^82^ compared tomato slices dried in open sun to those dried in a solar tunnel dryer and showed that the highest RR was noticed in the solar tunnel-dried slices due to the shorter drying time. Similarly, Rajkumar et al. ^81^, reported comparable findings, where the RR of tomato slices dried in a cabinet SD with slice thicknesses of 4, 6, and 8 mm were 3.25, 3.56, and 3.61, respectively, compared to the open sun-dried slices, which had RR values of 2.95, 3.15, and 3.24. This indicates that the highest RR was achieved in tomato slices dried in the cabinet SD, and the RR increased with greater slice thickness.

### Consequence of drying parameters on PH and acidity

**Fig. 12:**
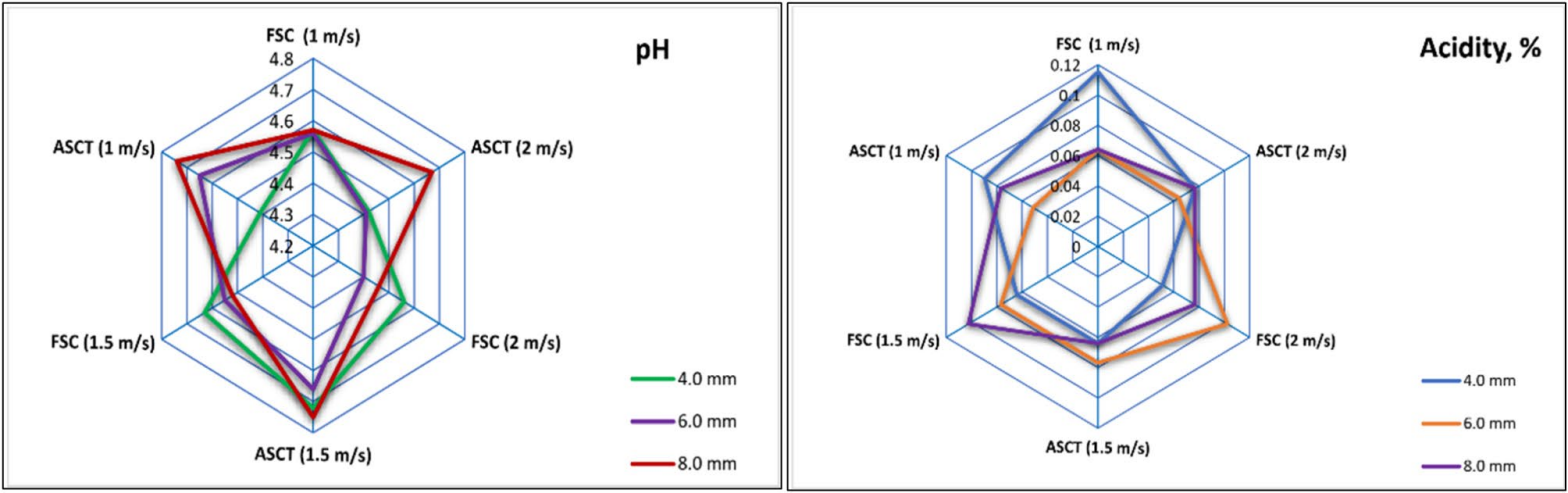
Consequence of draying parameters on pH and acidity of dried tomatoes.

The drying process caused an increase in the pH of dried tomato slices compared to fresh tomatoes. Specifically, the pH rose from 4.12 for fresh tomatoes to 4.75 for dried tomato slices. The highest pH values were observed in the dried tomato slices treated with ASCT, in contrast to those dried using FSC. Additionally, reducing the hot air velocity led to an increase in air temperature, which in turn raised the pH values of the dried tomato slices, as illustrated in Fig. 12. These findings are consistent with previous studies conducted by Babarinde et al., wh0ich dried tomatoes at various air temperatures of 50, 55, and 60 °C. Their results showed that the pH value increased with higher drying air temperatures.

Also, the findings align with the results reported by Elghazali et al. ^14,83^ and Elghazali et al. ^14,83^; where they observed a decrease in pH values after drying. In contrast, the acidity values of the dried tomato slices were lower than those of fresh tomatoes, as the drying process reduced acidity across all drying systems, air velocities, and slice thicknesses, as shown in Fig. 12. This is consistent with the findings of Toor and Savage ^84^; Babarinde et al. ^85^; Elghazali et al. ^14,83^ and Elghazali et al. ^14,83^. Nonetheless, Dufera et al. ^82^, demonstrated an increase in the acidity of dried tomatoes after the drying process.

### Influence of drying parameters on ascorbic acid (AA)

**Fig. 13:**
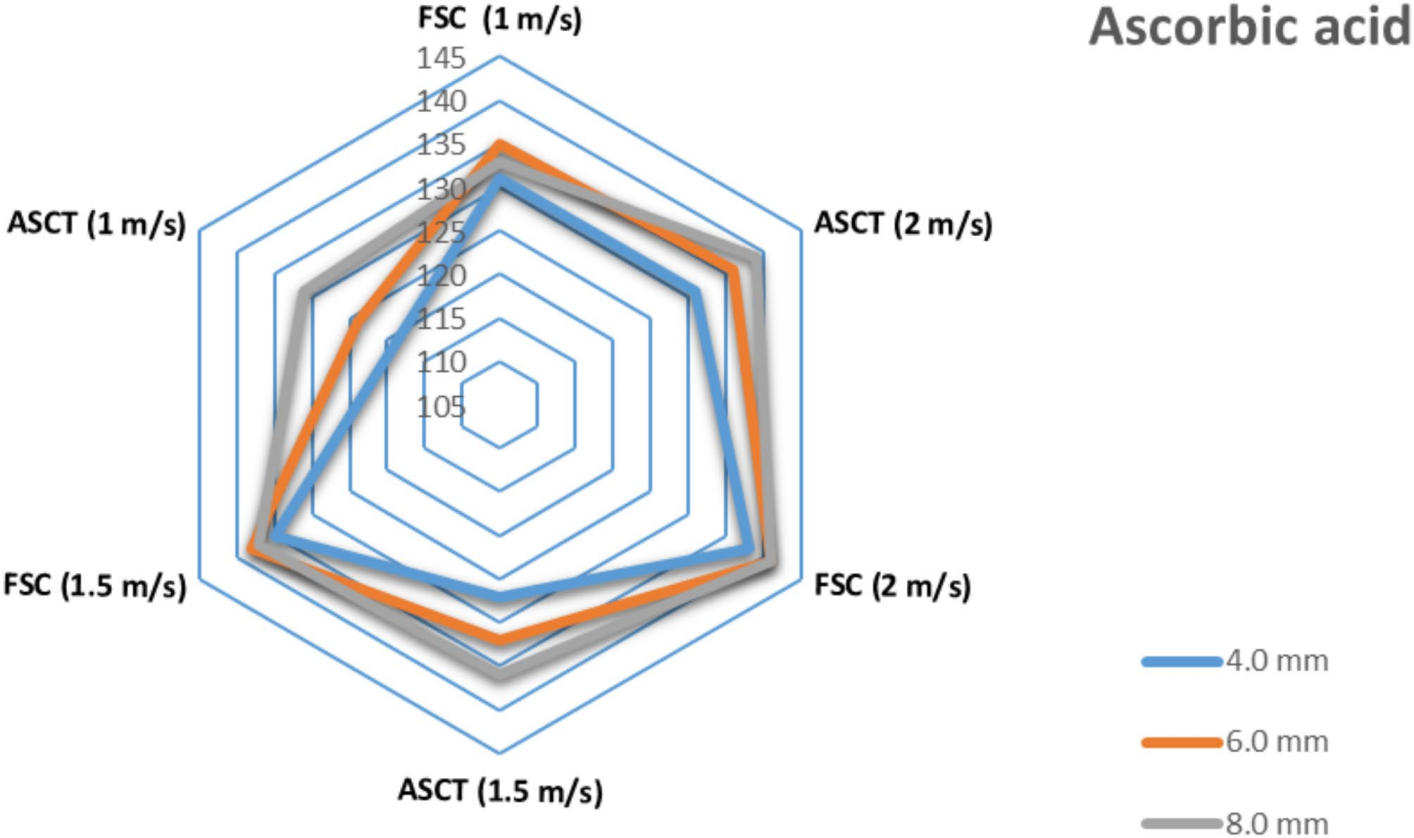
Impact of draying parameters on ascorbic acid content in dried tomatoes.

This study evaluated the effects of various drying techniques, hot air velocities, and slice thicknesses on the levels of AA in fresh and dried tomato slices. The AA content in fresh tomatoes decreases after the drying process, with significant variations observed among the dried tomato slices using different methods, air velocities, and slice thicknesses, as illustrated in Fig. 13. The highest AA content recorded was 141 mg/100 g (dry basis) in tomato slices that had an 8.0 mm thickness, dried using the SD method combined with FSC at an air velocity of 2 m/s. In contrast, a lower AA content of 131 mg/100 g (dry basis) was found in slices dried with SD integrated with ASCT at a 4.0 mm slice thickness and an air velocity of 1 m/s. This reduction may be attributed to the lower drying temperature used in that method. The retention of AA is affected by both air temperature and drying time, given AA’s high sensitivity to heat^86^. Dufera et al. ^82^ reported a similar reduction in AA concentration in fresh tomatoes after drying, with minimal AA loss occurring when the drying duration was brief and the temperature was low. Purkayastha et al. ^78^ also found that the greatest decrease in AA content happened at the maximum drying air temperature, with air velocity kept fixed at 1.1 m/s.

#### Influence of drying parameters on total phenols

**Fig. 14:**
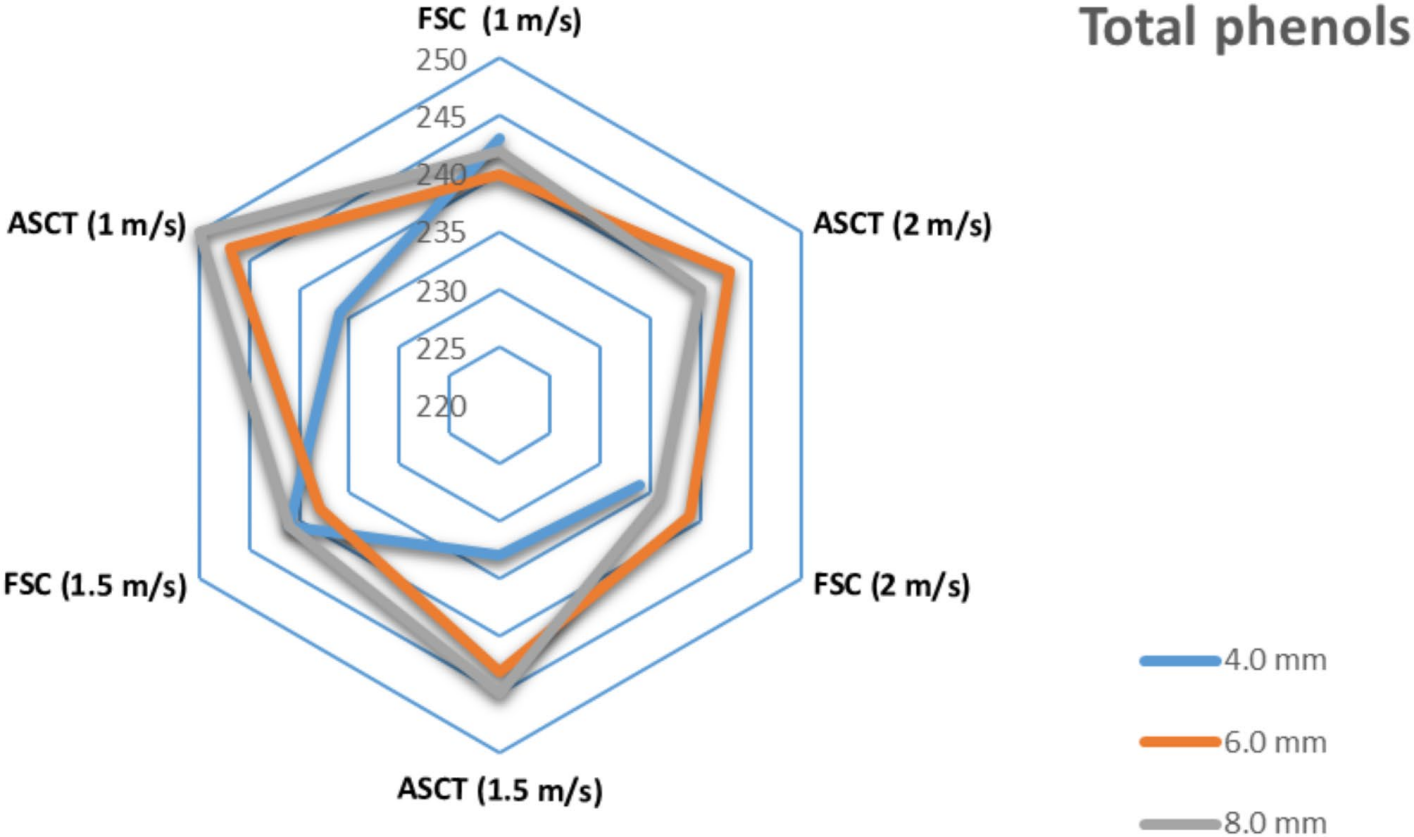
Impact of draying parameters on TP of dried tomatoes.

Figure 14 shows the total phenolic (TP) content of tomato slices dried using different drying systems, hot air velocities, and slice thicknesses. Fresh tomatoes had a TP content of 432 mg of GAE per 100 g of DM. After the drying process, the TP content increased in all dried tomato samples; however, it decreased with lower hot air velocities. Simar results were reported by Babarinde et al. ^82^, where drying significantly reduced the TP content of tomato slices. In that study, fresh samples had a TP content of 586.26 mg of GA/100 g DM, while solar tunnel-dried tomatoes ranged from 259.96 to 362.95 mg GA/100 g DM, and sun-dried tomatoes had 169.51 mg GA/100 g DM. The reduced phenolic content in SD integrated with FSC may be attributed to the prolonged drying time, which can degrade some phenolic compounds, as noted by Dufera et al. ^82,87^ and Azeez et al. ^82,87^. These obtained results come in agreement with Azeez et al**.** ^87^**;** Kim and Chin ^88^; Santos-anchez et al.^21^ and Azeez et al. ^87^.

Figure 14 shows that tomato slices dried using a combination of SD and the ASCT method had a higher TP content compared to those dried with SD integrated with FSC. The highest TP content observed was 250 mg of GA/100 g DM, found in tomato slices dried with ASCT at a thickness of 8.0 mm and an air velocity of 1 m/s. In contrast, the lowest TP content, recorded at 234 mg GA/100 g DM, was noted in tomato slices dried with FSC at a thickness of 4.0 mm and an air velocity of 2 m/s. Moreover, the loss of TP content during drying was 42.13% for ASCT at 8.0 mm thickness and 1 m/s air velocity, compared to a 45.83% loss for FSC at 4.0 mm thickness and 2 m/s air velocity. These findings are consistent with prior research by Putra et al. ^74^, which reported that tomatoes dried at higher temperatures using vacuum oven drying (50, 60, and 70 °C) had higher TP levels than those dried at lower temperatures, with an air velocity of 0.1 m/s. Similarly, Kim and Chin ^88^ noted an increase in TP content with higher drying temperatures, varying from 60 to 100 °C. Also, Santos-Sánchez et al. ^21^ found that the lowest TP content loss occurred when drying at higher temperatures. The higher TP levels in tomatoes dried at elevated temperatures may be owing to the increased release of phenolic compounds from the cell walls, as heat treatment disrupts the ester bonds between phenolic components and the cell walls ^87^.

## Conclusion, recommendations, and future work

The current study aimed to enhance the drying quality of tomato slices by integrating a solar dryer with an IoT-controlled solar collector tracker. A key feature of this dryer is its advanced control system, which uses IoT technology to track the sun, overcoming the limitations of fixed flat-plate solar collectors. This innovation resulted in higher and more stable air temperatures within the drying chamber throughout the day, reduced drying times, and improved the quality of the dried tomatoes. The solar dryer was tested at various air velocities ranging from 1.0, 1.5, and 2.0 m/s with tomato slice thicknesses between 4.0, 6.0, and 8.0 mm. The quality of the dried tomatoes was evaluated based on several criteria, including final moisture content, total phenol content, ascorbic acid, pH, color, acidity, and rehydration ratio. The results showed that the air temperature rose by about 18.43% in the drying room of the SD connected to the ASCT and the relative humidity dropped by about the same amount. This was in contrast to the other SD connected to the FSC, which had different air speeds. Additionally, the final MC decreased with decreasing slice thickness for tomato slices. Where the lowest MC was observed in slice thickness of 4 mm and at air velocity of 1.5 m/s, which means that drying tomatoes at slice thickness of 4 mm at 1.5 m/s air velocity reached the equilibrium moisture content faster than the other air speeds and slice thicknesses. Conversely, the color analysis of dried tomato slices revealed that the drying systems, slice thickness, and air velocities significantly influenced the final color values. Specifically, lightness (L*, a*, and b*) increased with higher air velocities, with ASCT producing greater values than FSC. The chemical analysis of dried tomato slices also revealed that the samples in ASDT had higher rehydration ratios, pH, and total phenols, but lower acidity and ascorbic acid. Overall, the combination of SD and ASCT proves to be a viable method for drying tomatoes, retaining a high level of the original quality. Further research is suggested to explore the microbial safety, antioxidant levels, and shelf life of ASCT-dried tomatoes, particularly when stored in various packaging materials.

## Data Availability

All data are provided within the article.

## List of symbols

*D*_*min*_: Average smallest diameter, mm
*D*_*max*_: Average greatest diameter, mm
*D*_*a*_: Athematic average diameter, mm
*D*_*g*_: Geometric average diameter, mm
*S*_*a*_: Surface area, mm^2^
*H*: Hight, mm
*L**: Lightness
*a**: Redness
*b**: Yellowness
$${W}_{f}$$: Final weight, g
$${W}_{i}$$: Initial weight, g

## Abbreviations

ASCT: Automatic solar collector tracker
FSC: Fixed solar collector
SC: Solar collector
MC: Moisture content
DP: Drying process
AD: Average diameter
GD: Geometric mean diameter
SD: Solar dryer
AA: Ascorbic acid
TP: Total phenols
RR: Rehydration ratio
IoT: Internet of things
PV: Photovoltaic
ICI: International commission on illumination
LDR: Light diode resistor

## Subscripts

a: Average
min: Minimum
max: Maximum
g: Geometric
in: Input
*out*: *Output*
oc: Open circuit
sc: Short circuit
f: Final
i: Initial

## Greek letters

$$\varphi$$: Sphericity
